# Authors’ Reply: “The Problem of Investigating Causal Relationships Between Cognitive and Evaluative Variables”

**DOI:** 10.2196/52877

**Published:** 2023-11-22

**Authors:** Bernhard Guetz, Sonja Bidmon

**Affiliations:** 1 Carinthia University of Applied Sciences Villach & Klagenfurt Austria; 2 Department of Marketing and International Management Alpen-Adria-Universitaet Klagenfurt Klagenfurt Austria

**Keywords:** social influence, physician rating websites, patient satisfaction, eHealth literacy

We appreciate the comments made by Konerding [1] and are thankful for the opportunity to take part in this research dialogue. As described in detail in our paper [2], the fact that social influence has an impact on the behavioral intention to use a technology has been postulated in numerous theories (eg, [3]), with the Unified Theory of Acceptance and Use of Technology (UTAUT) [4] being the most prominent one. This relationship has been tested and proven in many empirical studies (eg, [5,6]), usually by the application of a cross-sectional study design [7]. Thus, we applied this methodology to our research domain in line with the UTAUT research stream.

We are aware of the ongoing methodological debate about whether cross-sectional data are appropriate to test causal relations between variables. However, the application of a wide range of techniques for data collection and analysis has been the subject of continuing controversy in research for a long time [8,9]. In the past 3 years, the *Journal of Medical Internet Research* has published several studies applying a similar methodology to the one we have chosen (ie, creating a mediational model and testing it with cross-sectional data; eg, [10,11]). As Spector [8] notes, there is perhaps no research design that has been more used, albeit more maligned, than the cross-sectional design. He underlines, however, that the longitudinal design’s ability to reflect causality has been overstated and is not advantageous as compared to the cross-sectional design in most cases of use.

As Konerding [1] states, causal relationships between cognitive and evaluative variables are very difficult to investigate. However, we tried to resolve this challenge by manipulating the independent variable. Thus, we even went a step further than other studies [10,11] by applying a mixed methods approach including an experimental setting. Although variables such as the credibility of online portals probably cannot be manipulated meaningfully, other methods such as the think-aloud method, eye-tracking, or neuroscientific methods could be considered for related research projects in the future.

In his concluding sentence, Konerding [1] suggests giving adequate consideration to methodological limitations when interpreting results. In this context, we agree but also note that all possible limitations referring to the methodology used in our research endeavor have been addressed within the published paper. Additionally, Konerding [1] misstates the direction of influence in the proposed influencing paths (see the end of the second paragraph).

Last, we do not agree that the study by Maddux and Rogers [12] can be interpreted as a best practice example suitable for our research domain. We appreciate the authors’ theoretical contribution and merit but think that a sample size of 153 undergraduate students can be criticized regarding the weak representativeness and an increased probability of bias such as nonresponse [7,13]. Additionally, concerns about the inappropriate reliance on undergraduates have been raised since 1975 [14].

Taken together, we think that methodological discussions can be fruitful in general as long as they facilitate new ways of thinking.

## Abbreviations

UTAUT: Unified Theory of Acceptance and Use of Technology
